# Production and characterization of graphene oxide-engineered biochars and application for organic micro-pollutant adsorption from aqueous solutions

**DOI:** 10.1007/s11356-023-28549-y

**Published:** 2023-07-11

**Authors:** Panagiotis Regkouzas, Labrini Sygellou, Evan Diamadopoulos

**Affiliations:** 1 School of Chemical and Environmental Engineering, Technical University of Crete, 73100 Chania, Greece; 2 Institute of Chemical Engineering Sciences (ICE-HT), Foundation of Research and Technology, 26504 Patras, Rio Greece

**Keywords:** Biochar, Graphene oxide, Rice husks, Sewage sludge, Adsorption, Organic micro-pollutants

## Abstract

In this study, conventional and Graphene Oxide-engineered biochars were produced and thoroughly characterized, in order to investigate their potential as adsorptive materials. Two types of biomass, Rice Husks (RH) and Sewage Sludge (SS), two Graphene Oxide (GO) doses, 0.1% and 1%, and two pyrolysis temperatures, 400 °C and 600 °C were investigated. The produced biochars were characterized in physicochemical terms and the effect of biomass, GO functionalization and pyrolysis temperature on biochar properties was studied. The produced samples were then applied as adsorbents for the removal of six organic micro-pollutants from water and treated secondary wastewater. Results showed that the main factors affecting biochar structure was biomass type and pyrolysis temperature, while GO functionalization caused significant changes on biochar surface by increasing the available C- and O- based functional groups. Biochars produced at 600 °C showed higher C content and Specific Surface Area, presenting more stable graphitic structure, compared to biochars produced at 400 °C. Micro-pollutant adsorption rates were in the range of 39.9%-98.3% and 9.4%-97.5% in table water and 28.3%-97.5% and 0.0%-97.5% in treated municipal wastewater, for the Rice Husk and Sewage Sludge biochars respectively. The best biochars, in terms of structural properties and adsorption efficiency were the GO-functionalized biochars, produced from Rice Husks at 600 °C, while the most difficult pollutant to remove was 2.4-Dichlorophenol.

**Supplementary Information:**

The online version contains supplementary material available at 10.1007/s11356-023-28549-y.

## Introduction

Biochar is a carbonaceous solid product that can be produced by biomass pyrolysis or other carbonization processes (Ahmad et al. 2014; Zhao et al. 2021). One of the largest areas of biochar application concerns its environmental use as an adsorbent. High C content, large surface area and porosity, abundance of surface functional groups and other favorable physicochemical properties make biochar a candidate for organic or inorganic pollutant adsorption material from water or soil (Ahmed et al. 2016; Rajapaksha et al. 2016; Liu et al. 2015). These properties are directly affected primarily by biomass type and pyrolysis temperature and secondarily by pyrolysis residence time and heat rate (Li et al. 2017). Moreover, these properties can be further tuned in by adopting several modification techniques, like acid or base treatment (Wang et al. 2019; Sizmur et al. 2017), or by incorporating in the biochar matrix materials with specific properties, such as carbon nanostructures (graphene, graphene oxide, CNTs) (Tan et al. 2016; Liu et al. 2016), metal oxides (Li et al. 2018), or magnetic materials (Wang et al. 2019), in order to serve targeted applications and needs.

One of these nanomaterials is Graphene Oxide (GO), which is a 2-D graphene sheet that can be produced by using various chemical methods to oxidize graphite (Dimiev & Tour 2014). GO exhibits high surface chemical reactivity, by the existence of various C- and O- based functional groups and has good thermal and mechanical properties, due to its highly porous nature (De Marchi et al. 2018). The combination of GO with biochar can provide thermally stable products with greater properties and sorptive ability than the conventional biochars, even if used in small quantities (Zhang et al. 2012). For example, when GO is implemented into biochar, it improves biochar surface functional groups (Abdul et al. 2017), the specific surface area and pore volume (Ghaffar & Younis 2014) and its adsorption ability, mostly for the removal of organic contaminants (Huang et al. 2017; Moyo et al. 2020).

Recently, organic contaminants, like pharmaceuticals, pesticides, hormones, personal care products and illicit drugs have been increasingly being found both in the influent and effluent of WWTPs, as well as in surface waters. Despite the fact that these compounds are being found in relatively small concentrations, the potential damage they can provoke to the recipient environment (surface water and its living organisms) could be great and of unknown scale (Rathi & Kumar 2021). As a result, organic pollutants are being continuously monitored and studied, both for their occurrence and for the techniques available to treat them efficiently. Moreover, the potentially most dangerous and complicated organic pollutants are being monitored and included in the list of ‘Contaminants of Emerging Concern’, which is being updated and enriched on a yearly basis, due to its importance (Richardson & Kimura 2019; Palansooriya et al. 2019).

The use of waste biomass as feedstock for biochar production, combined with a relatively small dose of GO could lead to a sustainable, cost-effective and enhanced adsorptive material that could compete with other adsorptive materials and could be successfully applied as tertiary treatment of wastewater, in order to remove emerging pollutants, such as EDCs (Inyang & Dickenson 2015; Qiu et al. 2022). This application of biochar is favorable due to its sorptive capacity that is attributed to its porous nature, high surface area, carbonaceous structure and abundance of surface functional groups which play an active role concerning cost-effective organic or inorganic pollutant adsorption from water and wastewater (Jin et al. 2022). One of biochar main benefits is its ability to effectively remove these pollutants from wastewater even at low initial concentrations, like in realistic conditions (Qiu et al. 2021; Liu et al. 2023). As a result, the goal of this work was to produce GO functionalized biochars, using two waste biomasses, Rice Husks (RH), which is a carbon rich agronomic waste and Sewage Sludge (SS), which represents a difficult-to-manage, waste-originated biomass, at two pyrolytic temperatures (400 °C and 600 °C) and two GO doses (0.1% and 1%), in order to thoroughly characterize them and test them as sorptive materials for the adsorption of six organic micro-pollutants of emerging concern from water and wastewater samples. The novelty of this study concerns the extended physicochemical and structural characterization of GO-doped biochar nano-composites, combined with the realistic environmental application of the produced materials as adsorbents for EC removal from water and wastewater, in close-to-realistic initial pollutant concentrations, where usually in literature higher pollutant concentrations are being studied in order to provide easier and clearer trends concerning pollutant adsorption.

## Experimental

### Reagents

The reagents used in this work are described in the following list:

- Androsterone 97,7%, Sigma Aldrich, CAS Number: 53–41-8.
- Bisphenol A 99,5%, Dr Ehrenstorfer, CAS Number: 80–05-7.
- Estrone 99,3%, Sigma Aldrich, CAS Number: 53–16-7.
- Graphite powder, BAYCARBON Inc., Product SP-1.
- Hydrochloric Acid, Sigma Aldrich, CAS Number: 7647–01-0.
- Hydrogen Peroxide, Chem-Lab, CAS Number: 7722–84-1.
- Nitric Acid, Sigma Aldrich, CAS Number: 7697–37-2.
- 19-Norethindrone 99.6%, Sigma Aldrich, CAS Number: 68–22-4.
- Orthophosphoric Acid, Scharlau, CAS Number: 7664–38-2.
- Potassium Bromide, Fluka, CAS Number: 7758–02-3.
- Potassium Permanganate, Merck, CAS Number: 7722–64-7.
- Sulfuric Acid, Sigma Aldrich, CAS Number: 7664–93-9.
- Vanadium Pentoxide, Merck, CAS Number: 1314–62-1.
- 17α-Ethinylestradiol 99,5%, Dr Ehrenstorfer, CAS Number: 57–63-6.
- 2,4-Dichlorophenol 99,7%, Supelco, CAS Number: 120–83-2.

### Graphene oxide production

GO was produced by adopting a modified Hummers method (Hummers & Offeman 1958). Briefly, 12 g of graphite powder were added into a conical flask, along with 1440 mL of H_2_SO_4_ and 160 mL of H_2_PO_4_. The mix was stirred overnight at 300 rpm. Next, the flask was placed in an ice-bath, until the temperature dropped to 0 ± 2 °C. Then, 72 g of KMnO_4_ were gradually added for oxidation to take place. After approximately 4 to 5 h, the temperature rose to 20 ± 2 °C and 250 mL of H_2_O_2_ were added to neutralize excess KMnO_4_. The mix was centrifuged to retrieve the oxidized graphite, washed with 3.6% HCl and centrifuged again after 1d. The next step was the exfoliation process, where the sample was daily centrifuged and washed with 1L of ultrapure water for approximately 12 days, until the sample’s pH value was higher than 3.0. When the exfoliation process was completed, the produced GO was freeze-dried and stored in a dark airtight-sealed plastic container.

### Biochar production

Two biomass types were used as feedstock for biochar production: a. Anaerobically digested dewatered secondary sewage sludge (SS), collected from the Psyttalia Wastewater Treatment Plant, located in Athens, Greece. b. Rice husks (RH), collected from a rice mill in northern Greece. After being received, the samples were oven-dried at 90 °C for 48 h and then pulverized to a particle diameter < 0.5 mm. After the pre-treatment procedure was completed, the samples were placed in a dark environment, stored in airtight plastic containers.

Untreated biochar samples were produced using air-tight sealed crucibles in a muffle furnace (Linn High Therm), at two pyrolysis temperatures, i.e. 400 °C and 600 °C. Pyrolysis conditions were achieved by supplying 99% pure Nitrogen gas at a 200L h^−1^ rate and using a heat rate of 6°C min^−1^. Concerning the engineered GO biochar production, the dip-coating (Inyang et al. 2014) procedure was adopted. First, GO suspensions were prepared by adding 0.4 g GO in 400 mL double-distilled, de-ionized water (0.1% GO w/v) and 4 g GO in 400 mL double-distilled, de-ionized water (1% GO w/v). Next, the water samples were ultra-sonicated at 25 kHz for 1 h, in order to create GO suspensions. After this step, 40 g biomass was added to the suspensions and then homogenized at 800 rpm, using a mechanical stirrer. Samples were, finally, oven-dried at 80 °C for 48 h and then were pyrolyzed for biochar production, in the same fashion as described above for the untreated samples. Based on the created suspensions, the produced functionalized biochars were comprised of two GO doses i.e. 1%(w/w) and 10%(w/w). After pyrolysis, all biochar samples were washed for impurities removal, using de-ionized water, oven-dried and stored in dark airtight plastic containers, until further use.

### Biochar and GO characterization

A series of physicochemical analyses were performed in order to characterize the produced biochar samples. Biochar yield was calculated as the ratio of biochar to dry feedstock used for its production. Bulk density was determined using the analogue VDLUFA-Method A 13.2.1 (EBC 2012). Specific surface area was determined by using a NOVA 2200, Thermo Scientific Surfer gas sorption analyzer (samples were de-gasified at 200 °C), by applying the ΒΕΤ (Brunauer-Emmet-Teller) method. Ash content was determined by dry combustion in a muffle furnace at 750°C for 6 h (Rajkovic et al. 2012). C, N, H and S content was determined using a CHNS-O EA 3000 (EuroVector) elemental analyzer. O content was calculated as described in Eq.(A1), while Volatile Solids (VS) content was calculated as described in Eq.(A2).

A1 $$O \left[\%\right]=100\%-Ash \left[\%\right]-C [\%]-N [\%]-H [\%]-S [\%]$$

A2 $$VS \left[\%\right]=100\%-Ash [\%]$$

pH and electrical conductivity (EC) values were determined using a Crison Instruments multi-meter (micropH 2202), after shaking biochar with de-ionized water, in a 1:20 ratio, for 90 min (Rajkovic et al. 2012). The Point of Zero Charge (PZC) of the biochars was determined by applying the pH-drift method (Uchimiya et al. 2011). Biochar surface functional groups were determined by FT-IR analysis, using a Perkin Elmer Spectrum 1000, FT-IR spectrometer. Finally, metal composition of the biomass and biochar samples was determined using an ICP-MS analyzer, after acid-digestion with nitric acid. All the analyses were performed in triplicate, while standard errors ranged between 0.1% and 5% for all measurements.

Additionally, five samples, i.e. GO, RH_600, RH_GO1_600, SS_600 and SS_GO1_600 were selected to perform further analysis (XPS and Raman). The surface analysis measurements were performed in a UHV chamber (P ~ 5 × 10^−10^ mbar) equipped with a SPECS Phoibos 100-1D-DLD hemispherical electron analyzer and a non-monochromatized dual-anode Mg/Al x-ray source for XPS. The XP Spectra were recorded with MgKa at 1253.6 eV photon energy, combined with an analyzer pass energy of 15 eV, giving a Full Width at Half Maximum (FWHM) of 0.85 eV for Ag3d5/2 line. The analyzed area was of 1 cm diameter. The atomic ratios were calculated from the intensity (peak area) of the XPS peaks, weighed with the corresponding relative sensitivity factors (RSF) derived from the Scofield cross-section, taking into account the electron transport properties of the matrix. For spectra collection and treatment, including fitting, the commercial software SpecsLab Prodigy (by Specs GmbH, Berlin) was used. The peaks were fitted using a mix of Gauss-Lorentzian peak functions, after a Shirley type background removal. The samples were in powder form and pressed onto In rod. *Micro-Raman measurements* were obtained using the T-64000 model of Jobin Yvon (ISA-Horiba group) in the backscattering configuration. The excitation wavelength (514.5 nm) was provided by a DPSS laser (Cobolt Fandango TMISO laser). The laser power on the sample was 2 mW and was focused on the samples by a 50 × microscope objective. The scattered beam passed through an appropriate edge filter (for the removal of the strong elastically scattered photons) and directed into the slit of the monochromator in the single spectrograph configuration. Dispersion and detection of the Raman photons were done by a 600 grooves/mm grating and a two-dimensional charge–coupled device (CCD) detector (operating at 140 K), respectively.

Finally, Scanning Electron Microscope (SEM) analysis was performed (Zeiss SUPRA 35VP-FEG, operating conditions 5–20 kV) on the above-mentioned samples, i.e. GO, RH_600, RH_GO1_600, SS_600 and SS_GO1_600, along with samples RH400, RH_GO1_400, SS400 and SS_GO1_400, in order to obtain a better view on the micro-structure of the samples and to investigate whether GO was successfully integrated in the final functionalized biochar samples.

### Organic micro-pollutant adsorption experiments

Six organic micro-pollutants were selected for the conduction of the adsorption experiments; two phenols, 2-4D and BPA, two estrogens E1 and EE2, and two androgens, ADT and NOR. These compounds have been identified as Emerging Contaminants (ECs) and are being found in WWTP effluents, in concentrations of ng L^−1^ to μg L^−1^ (Palansooriya et al. 2019; Gogoi et al. 2018). Thus, the following initial concentrations were selected to produce the polluted mix: 2-4D and BPA: 20-40 μg L^−1^, E1 and EE2: 40-60 μg L^−1^, ADT and NOR: 70-90 μg L^−1^. The mentioned pollutants were spiked into 500 mL of table water, creating the initial polluted mix. Fresh pollutant mix was synthesized daily, in order to avoid photolytic/photocatalytic processes, which combined with the analytical methods adopted resulted to this initial pollutant concentration range. The adsorption experiments took place in light-protected conical flasks, at 22 ± 1℃, in duplicate repetition. 50 mL of the aqueous polluted sample and 0.15 g of biochar (equal to a dose of 3 g·L^−1^) were added in the flasks. Three adsorption residence times were investigated for each sample type, 10 min, 30 min and 60 min. After the adsorption experiments, samples were filtered with 0.45 μm PVDF Whatman filters. Additionally, four samples, i.e. RH600, RH_GO1_600, SS600 and SS-GO1_600 were selected to investigate their adsorptive behavior in a more realistic initial polluted mix, where the under investigation pollutants were spiked into 500 mL of secondary wastewater sample (before the chlorination stage), acquired from the Municipal Water & Sewerage Company of Chania (DEYAX). Information about the characteristics of both table water and wastewater samples are presented in Table S1 (Supplementary Material).

### Analytical methods for pollutant determination

The samples from the adsorption experiments were analyzed using Solid Phase Micro-Extraction followed by GC–MS analysis, according to a modified method developed by Antoniou et al., (2009). 10 mL of sample was inserted into an amber vial (15 mL, Supelco), which was airtight closed by means of a PTFE-silicone septum, along with 1.5 g NaCl. Sample pH was adjusted into the range of 2.5–3. The samples were vortexed for 1 min and then placed into a water-bath at 60 °C and 530 rpm for 1 h, while an SPME Fiber Assembly 85 μm Polyacrylate Fused Silica 24 GA, Supelco was inserted through the septum into the vial. After 1 h, the fiber was removed from the vial and injected into a Shimatzu GCMS-QP5050A (Gas chromatograph Mass Spectrometer), where desorption took place for 10 min at 290 °C and the analysis followed.

## Results & Discussion

### Biochar physicochemical characterization

Table 1 contains the results regarding biochar characterization, in terms of physicochemical properties. More specifically, it includes information about biochar yield, pH, electrical conductivity, point of zero charge, ash content and volatile matter, specific surface area and bulk density. The results showed that there were three main parameters that affected biochar properties: pyrolysis temperature, feedstock type and functionalization with GO. Biochar yield was in the range of 38.3–48.5% for the RH biochars, while for SS biochars biochar yield was slightly higher, ranging between 45.8% and 55.7%. Biochar yield was mainly affected by pyrolysis temperature, where biochars produced at 600 °C recorded lower yields than those produced at 400 °C by 10–11%, and secondarily by feedstock type, where SS biochars recorded 7–8% higher yields than RH biochars. It is well known that in higher temperatures, biochar yield decreases due to the increased loss of biomass volatile content, leading to elevated biochar inorganic content and to better organized C structures (Liu et al. 2015).pH values were in the range of 4.6–9.2 for the RH biochars and 6.8–8.6 for the SS biochars. Samples produced at the lower pyrolysis temperature showed slightly acidic to neutral pH values, while for the higher temperature, pH values were neutral to slightly alkaline. Biochar pH was affected mostly by pyrolysis temperature and by the functionalization process. Specifically, as mentioned above, higher pyrolysis temperature led to more alkaline pH values by 0.3–2.7 units, which is consistent to the findings of our previous work (Regkouzas & Diamadopoulos 2019). On the other hand, GO functionalization decreased RH biochar pH values by 0.7 to 2.5 units and SS biochar pH values by 0.9 to 1.1 units, which is attributed to the acidic pH of GO. Concerning the pH_PZC_ analysis, RH biochars indicated acidic to mostly neutral pH_PZC_ values, ranging between 4.4 and 7.2, while for the SS biochars pH_PZC_ values were within a higher range of 6.9–7.7. In most samples, pH_PZC_ values were lower than their respective pH values, while GO functionalization led to the decrease of pH_PZC_ values by 0.4–1.9 for the RH samples, while no significant change was observed for the SS biochars. Additionally, the higher pyrolysis temperature also led to higher pH_PZC_ values in the range of 0.3–1.2 for all samples, which has also been found in the past by other researchers (Uchimiya et al. 2011; Oh et al. 2012). Furthermore, EC values ranged from 101.7μS cm^−1^ to 190.5μS cm^−1^ for the RH biochars and from 199.3μS cm^−1^ to 592.7μS cm^−1^ for the SS biochars. The highest EC value was found in the SS_GO1_400 sample (i.e. 592.7μS cm^−1^), while the RH_GO1_600 sample recorded the lowest value (i.e. 101.7μS cm^−1^). Biomass type was the parameter that defined the most significant differences in the EC values. Specifically, the SS biochars recorded 70–80% higher values than the respective RH biochars. Moreover, GO functionalization and higher pyrolysis temperature caused decreased EC values for the RH samples, while the opposite trend was observed for the SS samples, where these two parameters increased biochar EC.

**Table 1:** Biochar physicochemical characterization. Values are means (triplicate repetition) ± standard deviation

Sample ID	Yield (%)	pH	pH_PZC_	EC (μS·cm^−1^)	Ash (%)	VS (%)	S_BET_ (m^2^·g^−1^)	Bulk Density (Kg·m^−3^)
RH_400	44.3 ± 1.8	6.5 ± 0.1	6.3	179.6 ± 3.2	37.8 ± 0.3	62.2 ± 0.4	59.4 ± 2.3	303 ± 19
RH_600	38.4 ± 1.4	9.2 ± 0.0	7.2	190.5 ± 3.7	45.6 ± 0.0	54.4 ± 0.1	214.9 ± 7.1	312 ± 8
RH_GO0.1_400	47 ± 1.1	5.8 ± 0.1	5.7	110.5 ± 2.6	40.8 ± 1.3	59.2 ± 1.6	47.5 ± 2.5	277 ± 3
RH_GO0.1_600	39.6 ± 0.7	8.5 ± 0.0	6.8	136.6 ± 5.8	45.9 ± 1.0	54.1 ± 1.2	234.2 ± 4.3	284 ± 8
RH_GO1_400	48.5 ± 0.7	4.6 ± 0.1	4.4	123.8 ± 5.0	34.3 ± 0.2	65.7 ± 0.3	86.2 ± 0.9	197 ± 1
RH_GO1_600	38.3 ± 0.6	6.7 ± 0.0	5.6	101.7 ± 2.8	42.4 ± 0.5	57.6 ± 0.6	211.8 ± 5.9	203 ± 3
SS_400	54.9 ± 0.4	7.9 ± 0.0	7.1	333.3 ± 5.0	45.7 ± 0.5	54.3 ± 0.6	14.3 ± 0.1	722 ± 8
SS_600	46.2 ± 0.4	8.6 ± 0.1	7.4	199.3 ± 2.1	64.1 ± 1.8	35.9 ± 2.2	52.5 ± 5.9	751 ± 2
SS_GO0.1_400	55.7 ± 1.3	6.9 ± 0.0	7.2	407.7 ± 5.7	54.7 ± 0.4	45.3 ± 0.5	21.0 ± 0.6	642 ± 2
SS_GO0.1_600	45.8 ± 0.5	7.8 ± 0.1	7.7	226.7 ± 3.5	71.1 ± 0.7	28.9 ± 1.0	69.3 ± 1.2	683 ± 9
SS_GO1_400	54.3 ± 0.1	6.8 ± 0.0	6.9	592.7 ± 2.1	53.2 ± 3.2	46.8 ± 4.5	18.8 ± 1.1	562 ± 14
SS_GO1_600	45.9 ± 0.3	7.9 ± 0.0	7.5	326.3 ± 4.2	60.4 ± 0.4	39.6 ± 0.5	55.2 ± 2.6	530 ± 5

Ash content was in the range of 34.3%-45.9% for the RH biochars, while elevated values were found in the SS biochars, ranging between 45.7% and 71.1%. The exactly opposite results were observed for the VS values, which is normal since the ash content is directly connected to VM content through Eq. (A2). Specifically, VM content was in the range of 54.1%-65.7% for the RH biochars and 28.9%-54.3% for the SS biochars. Biochar ash content and VM were mainly affected by pyrolysis temperature and biomass type. Biochars produced at 600 °C presented the highest ash and lowest VM contents, which is attributed to the carbonization process and the increase of biochar mineral content, when produced at higher temperatures (Zhang et al. 2020). Moreover, SS biochars recorded higher ash values, due to the higher inorganic content of SS biomass (Oh and Seo 2016). Biochar bulk density and specific surface area (S_BET_) were in the range of 197-312 kg m^−3^ and 47.5-234m^2^ g^−1^ respectively for the RH biochars, while concerning the SS biochars, values were in the range of 530-751 kg m^−3^ and 14.3–69.3m^2^ g^−1^ respectively. These results proved the physicochemical superiority of the RH biochars compared to the SS biochars. Biochar bulk density was mainly affected by biomass type, where RH biochars recorded lower values by more than 50%, and secondarily by pyrolysis temperature, where higher temperature resulted in slightly higher bulk density values. Higher temperatures result into more porous and with improved surface materials (Tripathi et al. 2016), which was reflected in the results of the specific surface area of our samples, where RH biochars, produced at 600 °C, showed the highest S_BET_ values. Specifically, these samples had more than three times higher S_BET_ values, compared to the respective SS biochars. It should also be mentioned that GO-enriched biochars did not affect significantly biochar bulk density and S_BET_ values, which can be attributed to the small chosen GO doses (0.1% and 1%). S_BET_ is one of the main parameters that can be used to foresee the adsorption ability of biochars, where higher values provide more efficient adsorption potential (Ahmad et al. 2014). When compared to other biochar samples in literature (Abdul et al. 2017; Regkouzas & Diamadopoulos 2019; Chu et al. 2019), RH biochars presented good surface area values, but they could not reach the standards of activated carbons (Swat et al. 2017; Kong et al. 2019). This, however, does not mean that they will not be able to perform well as adsorptive materials.

Table 2 contains the results of the elemental analysis that was performed on the biochar samples. The main element of interest, C, was in the range of 38.1%-48.2% for the RH biochars and 23.1%-30.9% for the SS biochars. RH biochars produced at 600 °C had higher C content by up to13.2%, compared to those produced at 400 °C, while the opposite trend was observed for the SS biochars, where samples produced at 400 °C contained up to 21.7% more C than those produced at 600 °C. The best biochar in terms of C content was RH_GO1_600. Generally, higher pyrolysis temperature is expected to provide biochars with higher C content, since better organized C layers are formed and biochars gain a more aromatic C nature (Rajapaksha et al. 2016). This was indeed confirmed by the RH biochars. The ambiguous results concerning the SS biochars, which have been previously observed by other researchers (Wang et al. 2019; Oh et al. 2012; Kong et al. 2019; Jin et al. 2016), are attributed to the high ash content of sewage sludge, which affects the carbonization process, resulting in lower C content at higher pyrolysis temperatures (Fan et al. 2020). Additionally, GO functionalization slightly improved C content, but at very low percentages. Concerning the rest of the elements, H was found in small quantities in all samples (0.7%-2.1%), S was found at even smaller levels not exceeding 1.5%, while N was found in the range of 0.4%-0.6% for the RH biochars and 2.6%-3.8% for the SS biochars, which is attributed to the higher N content in the SS feedstock. Pyrolysis temperature affected these elements in the same fashion as in the C measurements. Biochar O content was calculated as shown in Eq. (A1). RH biochars contained 7.5%-21.8% O, while SS biochars showed slightly lower values, ranging between 6.2% and 16.6%. Higher pyrolysis temperature provoked lower O contents for both RH and SS biochars by up to 59.2%. This was expected, since the main variable that affects Eq. (A1) is biochar ash (Zheng et al. 2019). Moreover, O/C and H/C ratios were calculated from the mean values of the elements shown in Table 2. RH biochar O/C and H/C contents were in the range of 0.12–0.43 and 0.22–0.57 respectively, while SS biochars scored 0.20–0.40 and 0.36–0.82 accordingly. Both parameters were found to be affected by higher pyrolysis temperature, where the ratios were reduced by 14.2%-51.2%. This is normal, since at higher temperatures, the organic part of biomass is more successfully converted into well-carbonized condensed carbon moieties of a more aromatic nature (Abdul et al. 2017; Chu et al. 2019). As an overall conclusion of the results presented above, pyrolysis temperature is the main parameter that affects the physicochemical nature of biochars.

**Table 2:** Biochar elemental composition. Values are means (triplicate repetition) ± standard deviation

Sample ID	C (%)	H (%)	N (%)	S (%)	O (%)	O/C	H/C
RH_400	38.1 ± 0.3	1.8 ± 0.1	0.5 ± 0.0	BDL	21.8	0.43	0.57
RH_600	43.9 ± 0.1	1.1 ± 0.1	0.5 ± 0.0	BDL	8.9	0.15	0.30
RH_GO0.1_400	41.5 ± 2.1	1.9 ± 0.1	0.5 ± 0.0	0.1 ± 0.0	15.2	0.27	0.55
RH_GO0.1_600	42.9 ± 0.6	1.1 ± 0.1	0.4 ± 0.1	BDL	9.7	0.17	0.31
RH_GO1_400	45.4 ± 0.2	1.5 ± 0.0	0.6 ± 0.0	0.4 ± 0.0	17.8	0.29	0.40
RH_GO1_600	48.2 ± 3.9	0.9 ± 0.0	0.6 ± 0.0	0.4 ± 0.1	7.5	0.12	0.22
SS_400	30.9 ± 1.6	2.1 ± 0.0	3.8 ± 0.0	0.9 ± 0.0	16.6	0.40	0.82
SS_600	24.2 ± 0.2	0.8 ± 0.0	3.0 ± 0.0	0.9 ± 0.0	7	0.22	0.40
SS_GO0.1_400	26.9 ± 1.3	1.8 ± 0.0	3.7 ± 0.1	1.2 ± 0.1	11.7	0.33	0.80
SS_GO0.1_600	23.1 ± 0.8	0.7 ± 0.0	2.7 ± 0.1	0.9 ± 0.0	6.2	0.20	0.36
SS_GO1_400	30.8 ± 0.3	1.8 ± 0.0	3.7 ± 0.0	1.5 ± 0.1	11.7	0.28	0.70
SS_GO1_600	26.5 ± 1.9	0.8 ± 0.0	2.6 ± 0.1	1.2 ± 0.2	8.5	0.24	0.36

*BDL: Below Detection Limit

### Biochar metal composition

Results concerning the metal composition of biochars are presented in Table 3. SS biochars had a significantly larger metal content, which was expected, since sewage sludge is an anthropogenic waste, known to contain traces of metals in its structure. On the other hand, rice husks constitute an agronomic lignocellulotic waste, which is not expected to contain many metals, nor in large amounts. Specifically, K and Ca were the dominant metals in RH biochar structure, found in doses ranging from 1857 mg kg^−1^ to 2214 mg kg^−1^ and 520 mg g^−1^–1446 mg kg^−1^ respectively, followed by Mn, Mg and Al. The rest of the metals were found at much lower levels. SS biochars were significantly richer in terms of metal content, where Ca and Al were found in the highest quantity, ranging from 47081 mg kg^−1^ to 64791 mg kg^−1^ and 6911 mg g^−1^–9885 mg kg^−1^ respectively. Mg, Fe, K and Na were also found in significant quantity in the SS biochars. GO functionalization and pyrolysis temperature did not appear to affect biochar metal content. In some cases, higher pyrolysis temperature slightly raised the metal content of biochars, but not in a significant manner. Buss et al., (2016) stated that the effect of pyrolysis temperature on biochar metal content is strongly dependent on the physicochemical structure of the feedstock. It should also be highlighted that heavy metal (Zn, Cu, Ni, Cd, Pb, Hg, and Cr) values of all biochars did not exceed the limitations set by the European Union (Directive 86/278/EEC) for land application of sewage sludge. In addition to that, in our previous work it was shown that conversion of sewage sludge to biochar could be an effective method for the valorization of this waste, since although these biochars were rich in metal content, their leaching potential was significantly limited (Regkouzas & Diamadopoulos 2019). Other researchers also confirmed that conversion of biomass to biochar may increase the material’s metal content, but significantly limits its leaching potential (Phoungthong et al. 2018; Mendez et al. 2012).

**Table 3:** Biochar metal composition. Values are means (triplicate repetition) ± standard deviation

Metal	RH_400	RH_600	RH_GO0.1_400	RH_GO0.1_600	RH_GO1_400	RH_GO1_600	SS_400	SS_600	SS_GO0.1_400	SS_GO0.1_600	SS_GO1_400	SS_GO1_600
	mg metal · kg^−1^ sample											
B	2.7 ± 0.1	2.3 ± 0.1	3.3 ± 0.4	3.5 ± 0.8	4.8 ± 0.0	5.8 ± 0.2	8.8 ± 0.0	8.2 ± 0.9	15 ± 5	11 ± 0	10 ± 0	10 ± 1
Na	82 ± 31	60 ± 5	156 ± 48	123 ± 17	120 ± 1	177 ± 71	700 ± 8	890 ± 102	1186 ± 323	1017 ± 49	894 ± 43	887 ± 15
Mg	470 ± 5	277 ± 20	562 ± 91	416 ± 99	490 ± 1	606 ± 4	3838 ± 7	4423 ± 505	4947 ± 723	5273 ± 22	3811 ± 129	4275 ± 71
Al	100 ± 20	128 ± 14	110 ± 13	145 ± 21	145 ± 3	151 ± 5	7293 ± 116	8074 ± 844	8985 ± 1328	9885 ± 47	6911 ± 141	7612 ± 213
Si	BDL	BDL	BDL	BDL	BDL	BDL	BDL	BDL	BDL	BDL	BDL	BDL
K	2074 ± 83	2136 ± 86	2078 ± 269	2109 ± 259	2214 ± 3.8	1857 ± 75	992 ± 8	1181 ± 128	1390 ± 342	1349 ± 34	1081 ± 49	1170 ± 13
Ca	833 ± 225	520 ± 97	1371 ± 243	1446 ± 510	663 ± 54	1027 ± 9	51,945 ± 411	55,461 ± 6504	60,027 ± 7967	64,791 ± 206	47,081 ± 717	51,976 ± 1806
Cr	1.2 ± 0.4	0.9 ± 0.1	1.1 ± 0.2	1.0 ± 0.2	1.2 ± 0.0	1.3 ± 0.0	237 ± 5	191 ± 19	265 ± 33	243 ± 2	219 ± 4	192 ± 7
Mn	202 ± 12	161 ± 8	263 ± 19	206 ± 31	315 ± 1	402 ± 1	102 ± 1	107 ± 11	112 ± 15	119 ± 1	129 ± 2	145 ± 5
Fe	44 ± 1	73 ± 12	103 ± 12	145 ± 56	123 ± 1	134 ± 3	2862 ± 82	3027 ± 175	2710 ± 409	2713 ± 7	2200 ± 5	2355 ± 98
Co	BDL	BDL	BDL	BDL	BDL	BDL	0.4 ± 0.0	0.4 ± 0.2	0.7 ± 0.2	0.8 ± 0.1	0.4 ± 0.0	0.5 ± 0.0
Ni	0.6 ± 0.2	2.1 ± 0.5	0.5 ± 0.1	1.8 ± 0.2	0.9 ± 0.1	1.9 ± 0.1	34 ± 1	28 ± 4	41 ± 7	34 ± 1	33 ± 1	29 ± 1
Cu	4.5 ± 0.0	4.6 ± 0.2	21 ± 1	36 ± 4	15 ± 1	17 ± 1	225 ± 2	205 ± 17	225 ± 22	227 ± 1	188 ± 1	194 ± 7
Zn	31 ± 8	24 ± 2	84 ± 4	86 ± 3	124 ± 0	151 ± 5	705 ± 2	688 ± 63	827 ± 92	858 ± 1	704 ± 9	717 ± 15
As	0.2 ± 0.0	0.1 ± 0.0	0.3 ± 0.0	0.2 ± 0.0	0.3 ± 0.0	0.3 ± 0.0	1.9 ± 0.0	1.7 ± 0.1	2.1 ± 0.2	1.9 ± 0.0	1.8 ± 0.0	1.7 ± 0.0
Se	BDL	BDL	BDL	BDL	BDL	BDL	0.3 ± 0.0	0.1 ± 0.0	0.6 ± 0.2	0.5 ± 0.0	0.4 ± 0.0	0.2 ± 0.0
Mo	0.2 ± 0.0	0.3 ± 0.0	0.3 ± 0.0	0.5 ± 0.0	0.3 ± 0.0	0.6 ± 0.0	4.9 ± 0.0	4.7 ± 0.5	6.0 ± 0.8	5.2 ± 0.0	5.4 ± 0.1	5.3 ± 0.0
Cd	BDL	BDL	BDL	BDL	BDL	BDL	0.9 ± 0.0	0.9 ± 0.1	1.0 ± 0.2	1.0 ± 0.0	0.9 ± 0.0	0.8 ± 0.1
Hg	0.9 ± 0.1	0.6 ± 0.0	0.7 ± 0.0	0.7 ± 0.1	0.7 ± 0.0	0.7 ± 0.0	0.5 ± 0.0	0.7 ± 0.1	0.4 ± 0.1	0.3 ± 0.0	0.4 ± 0.0	0.4 ± 0.1
Pb	0.6 ± 0.3	0.6 ± 0.2	2.0 ± 0.0	2.1 ± 0.2	1.9 ± 0.1	2.2 ± 0.2	148 ± 1	148 ± 17	149 ± 1	158 ± 1	139 ± 1	145 ± 1

*BDL: Below Detection Limit

### Biochar FTIR analysis

**Fig. 1:**
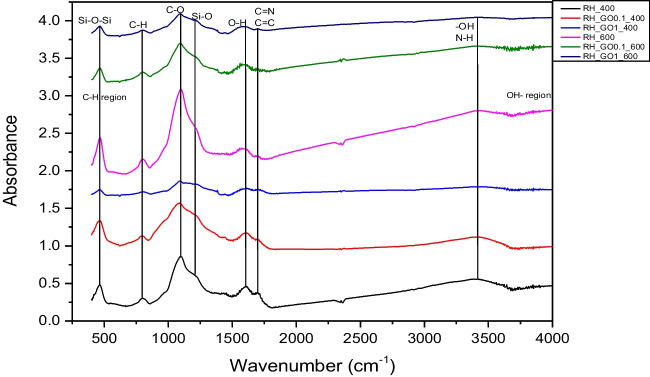
FTIR spectra of RH biochars

Figure 1 depicts the results of the FTIR analysis on the RH biochars. This analysis was performed to qualitatively determine biochar surface functional groups, which is valuable information, especially when biochar is studied as an adsorbent. RH biochars consisted of various functional groups, where C-O (1038 cm^−1^), C = N and C = C (1599 cm^−1^) were the dominant ones. Other functional groups that were found on RH biochar surface were C-H groups (887 cm^−1^), Si–O-Si (1038 cm^−1^) and Si–O (1100 cm^−1^) groups, O–H (1544 cm^−1^), as long as several –OH and amide groups in the region of 3440-3700 cm^−1^. It should be noted that the biggest vibrations, especially for C-O and –OH groups, were observed for samples produced at 600 °C, which confirms that higher pyrolysis temperatures provide better carbonization of biochar (Ahmed et al. 2016; Rajapaksha et al. 2016), as shown in the elemental analysis.

**Fig. 2:**
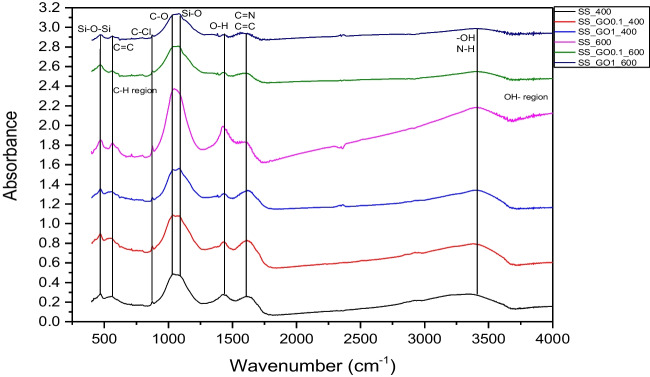
FTIR spectra of SS biochars

Similar functional groups were also found on SS biochar surface, as shown in Fig. 2. C-O (1038 cm^−1^), C = N (1599 cm^−1^) and C = C (665 cm^−1^ and 1599 cm^−1^) were the dominant groups in this case too, yet it can be observed that peaks at 1599 cm^−1^ were bigger compared to RH biochars. This is attributed to the N compounds in SS biochar, that provide stronger C = N functional groups. Although it is expected that SS biochars have more N-containing functional groups (Fan et al. 2020; Hossain et al. 2011), due to its mineral composition, this was not clear in this work. Moreover, -OH and –O functional groups were also found in higher quantity, compared to RH biochar. Other functional groups that were observed on SS biochar surface were C-H groups (887 cm^−1^), Si–O-Si (1038 cm^−1^), C–Cl (800 cm^−1^), Si–O (1100 cm^−1^) and O–H (1544 cm^−1^) groups.

### Raman, XPS and SEM analyses on selected samples

In order to attain a spherical view over the graphitic structure of our samples, additional analyses, Raman and XPS, were performed on selected samples, i.e. GO, RH600, RH_GO1_600, SS600 and SS_GO1_600. Additionally, SEM analysis was performed at the before-stated samples, along with biochars produced at 400℃, i.e. RH400, RH_GO1_400, SS400 and SS_GO1_400.

**Fig. 3:**
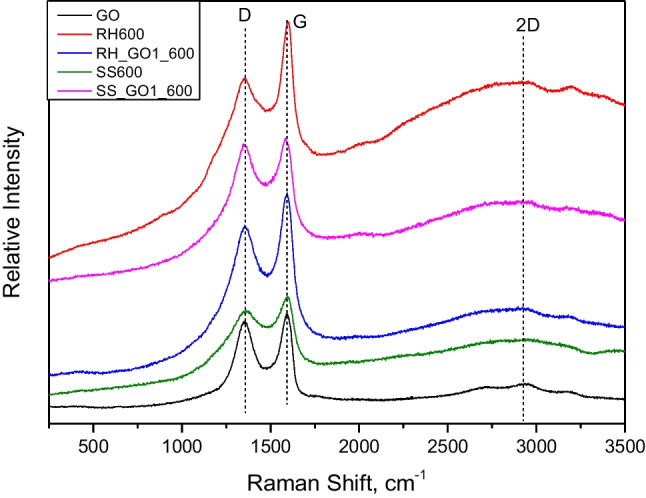
Raman spectra of selected samples

The results of the Raman analysis are presented in Fig. 3. Two dominant peaks were observed at 1359 cm^−1^ and 1594 cm^−1^ that correspond to the D band (sp^3^ hybridized C) and to the G band (sp^2^ hybridized C) (Abdul et al. 2017). Among the tested samples, important differences were observed for the D band and especially for the G band. Specifically, in RH biochars it was clear that the G band was stronger than the D band, implying different graphitic structure, compared to the SS biochars. Moreover, reduced peak intensity was observed for both D and G bands in the SS biochars, compared to RH biochars, which was also expected, since SS biochars have almost half of the C content, as shown in the elemental analysis. GO functionalization did not seem to significantly affect the Raman spectra of biochars, making biomass type the main parameter that affects the graphitic nature of biochar in our experiment. Finally, a third peak was observed at 2925 cm^−1^, corresponding to the 2D band (Zhang et al. 2018), which can provide information about the quantity of graphene sheet layers. The fact that in all samples small and narrow peaks were observed suggests that there may be stacked graphene/graphitic sheets in the samples’ core.

**Fig. 4:**
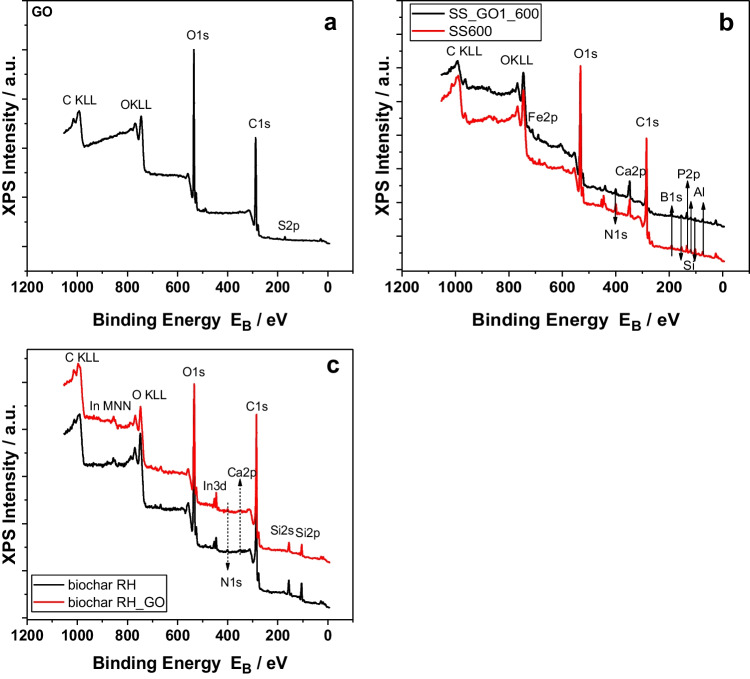
XPS survey scans of the investigated samples. **a**: Graphene Oxide (GO), **b**: SS biochars, **c**: RH biochars

Figure 4 presents the XPS survey scans of the investigated samples. Figure 4a refers to the results of GO, where two main atoms (C and O) were detected, along with some small traces of S, which could be attributed to the use of H_2_SO_4_ during GO production. Quantification in terms of atomic concentration is presented in Table 4. C was the main atom detected on GO surface at a percentage equal to 69.59%, while O was found at 30.41%. The absence of any other atoms was expected, since GO is a nano-scale carbonaceous structure, manufactured by graphite oxidation (Dimiev & Tour 2014).

**Table 4:** % atomic concentration and relative atomic ratios on GO surface

Peak	Eb [eV]	ASSIGNEMENTS	% ATOMIC C	RAR*
C1s	286.77	C–C	69.59 ± 0.02	1
`O1s	532.60	Mainly C-O	30.41 ± 0.02	0.44

*RAR: Relative Atomic Ratio

Figure 4b depicts the survey scan of the SS biochars, where the presence of C, O, P, Al, Si, N, Ca, B and Fe atoms was detected on biochar surface (Melia et al. 2018). The presence of multiple atoms can be confirmed by the ICP-MS and elemental analysis, where these atoms were detected in the highest quantity. It also shows that these elements are located both in biochar core and surface, creating various functional groups. Table 5 contains the necessary information about the atomic concentration of these atoms on SS600 and SS_GO1_600 surface. C and O were the main atoms detected in both biochar samples, in percentages equal to 32.33% and 34.06% respectively for SS600, while SS_GO1_600 had more C (53.50%) and less O (24.40%), which makes the engineered biochar better in physicochemical terms, since it contains more C-based functional groups on its surface, making it stronger as an adsorptive material (Zhang et al. 2018; Melia et al. 2018). Other atoms found on the surface of SS biochars were Al (6.13% and 4.70%), B (10.16% and 4.82%), P (6.07% and 2.88%) and Ca (4.48% and 2.37%).

**Table 5:** % atomic concentration and relative atomic ratios on SS600 and SS_GO1_600 surface

			SS600		SS_GO1_600	
Peak	Eb [eV]	ASSIGNEMENTS	% ATOMIC C	RAR*	% ATOMIC C	RAR*
C1s	284.90	C–C	32.33 ± 0.10	1	53.50 ± 0.08	1
Ca2p	347.72	Ca_3_(PO_4_)_2_	4.48 ± 0.02	0.14	2.37 ± 0.02	0.044
O1s	532.05	Mainly C-O	34.06 ± 0.08	1.05	24.40 ± 0.04	0.46
Al2p	74.77	Al_2_O_3_	6.13 ± 0.08	0.19	4.70 ± 0.05	0.089
Si2p	103.24	SiO_2_	3.11 ± 0.05	0.10	3.34 ± 0.04	0.062
P2p	133.89	Ca_3_(PO_4_)_2_, CaHPO_4_	6.07 ± 0.04	0.19	2.88 ± 0.03	0.054
B1s	191.29	N.D.**	10.16 ± 0.13	0.31	4.82 ± 0.08	0.09
Fe2p3/2	~ 712	FeOOH, Fe_2_O_3_	0.31 ± 0.01	0.01	0.20 ± 0.00	0.004
N1s	400.41	N–C	3.36 ± 0.03	0.10	3.79 ± 0.02	0.071

*RAR: Relative Atomic Ratio, **N.D.: not determined

Figure 4c shows the survey scan of the RH biochars. Fewer atoms were detected in these samples, when compared to the SS biochars, which was attributed, as stated above, to the feedstock biomasses. C, Ca, O, Si and N atoms were detected in both biochars. The main atoms were C and O in this case too, presenting similar trends regarding the C/O ratio to the SS biochars. Specifically, RH600 had 34.77% C and 44.05% O, while RH_GO1_600 had 66.37% C and 23.57% O, as presented in Table 6. This suggests that regarding the RH biochars, GO functionalization led to even better engineered biochar in physicochemical terms, having almost 29% more C on their surface. The rest of the atoms were detected in smaller quantities, except Si, which was detected at 19.27% for RH600 and 8.87% for RH_GO1_600.

**Table 6:** % atomic concentration and relative atomic ratios on RH600 and RH_GO1_600 surface

			RH600		RH_GO1_600	
Peak	Eb [eV]	ASSIGNEMENTS	% ATOMIC C	RAR*	% ATOMIC C	RAR*
C1s	285.16	C–C	34.77 ± 0.04	1	66.37 ± 0.05	1
Ca2p	349.15	Ca_3_(PO_4_)_2_	0.13 ± 0.01	0.004	0.18 ± 0.01	0.003
O1s	534.31	Mainly C-O	44.05 ± 0.04	1.27	23.57 ± 0.03	0.36
Si2p	104.91	SiO_2_	19.27 ± 0.05	0.55	8.87 ± 0.04	0.13
N1s	400.61	N–C	1.79 ± 0.04	0.051	1.03 ± 0.02	0.016

*RAR: Relative Atomic Ratio

**Fig. 5:**
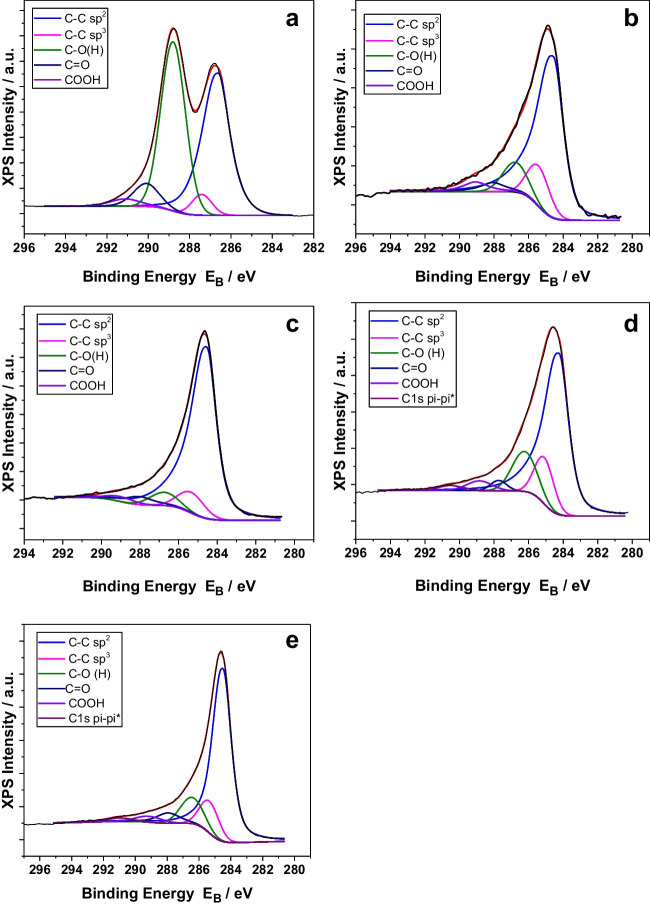
Deconvoluted C1s peaks of **a**: GO, **b**: SS600, **c**: SS_GO1_600, **d**: RH600, **e**: RH_GO1_600

Figure 5 contains the results regarding the deconvoluted C1s peaks of the samples under investigation, while Table 6 provides their quantification. Figure 5a concerns the GO sample, where the C1s peak shape is characteristic for GO samples. The C1s peak was deconvoluted into five components assigned to C–C with sp^2^ configuration, C–C with sp^3^ configuration, C-O(H), C = O and COOH bonds, as described in detail elsewhere (Sygellou et al. 2016). Figure 5b and c show the results that concerns biochars SS600 and SS_GO1_600 respectively. SS biochar C1s peaks were deconvoluted into the same five components, as the GO sample. Interesting information can be derived by the quantification of the different components, shown in Table 7, where the main components of SS biochars were C–C bonds with sp^2^ configuration (69.67% and 80.50%), followed by C–C bonds with sp^3^ configuration (11.78% and 7.77%), C-O (H) bonds (9.78% and 5.26%), C = O bonds (4.34% and 4.47%) and COOH bonds (4.42% and 1.99%). The main component that was affected by GO functionalization were the C–C bonds with sp^2^ configuration, where SS_GO1_600 had almost 11% more of this component than the non-functionalized SS600 sample, while the rest components concentration did not change significantly. Finally, Fig. 5d and e show the results of biochars RH600 and RH_GO1_600. RH600 and RH_GO1_600 biochar C1s peaks deconvoluted into six components, i.e. C–C bonds with sp^2^ configuration (66.68% and 69.58%), C–C bonds with sp^3^ configuration (12.07% and 10.50%), C-O (H) bonds (13.58% and 10.51%), C = O bonds (2.30% and 4.95%), COOH bonds (3.41% and 2.67%) and pi-pi* bonds (1.97% and 1.79%). As in the SS biochars, GO functionalization only affected the main component (C–C bonds with sp^2^ configuration), but in a smaller extent. Moreover, it is clear that in RH biochars, components are more evenly distributed, recording higher percentages in all components, compared to the SS biochars. This could mean that RH biochars would be better as adsorptive materials, due to their greater variety in C1s bonds on their surface.

**Table 7:** % component concentrations of C1s peaks

SS600			
Peak	Eb (eV)	% Component C	Assignement
C1s	284.58	69.67 ± 1.90	C–C sp^2^
C1s	285.43	11.78 ± 1.67	C–C sp^3^
C1s	286.78	9.78 ± 0.66	C-O(H)
C1s	288.08	4.34 ± 0.59	C = O
C1s	289.08	4.42 ± 1.15	COOH
SS_GO1_600			
Peak	Eb (eV)	% Component C	Assignement
C1s	284.54	80.50 ± 1.29	C–C sp^2^
C1s	285.34	7.77 ± 0.49	C–C sp^3^
C1s	286.74	5.26 ± 0.85	C-O(H)
C1s	288.04	4.47 ± 1.14	C = O
C1s	289.04	1.99 ± 0.28	COOH
RH600			
Peak	Eb (eV)	% Component C	Assignement
C1s	284.24	66.68 ± 0.72	C–C sp^2^
C1s	285.09	12.07 ± 0.18	C–C sp^3^
C1s	286.24	13.58 ± 0.47	C-O(H)
C1s	287.74	2.30 ± 0.24	C = O
C1s	288.84	3.41 ± 0.84	COOH
C1s	290.52	1.97 ± 0.17	pi-pi*
RH_GO1_600			
Peak	Eb (eV)	% Component C	Assignement
C1s	284.49	69.58 ± 1.71	C–C sp^2^
C1s	285.34	10.50 ± 0.51	C–C sp^3^
C1s	286.44	10.51 ± 1.50	C-O(H)
C1s	287.94	4.95 ± 1.51	C = O
C1s	289.14	2.67 ± 0.62	COOH
C1s	291.02	1.79 ± 0.18	pi-pi*

The results of the XPS analysis are similar to other works in literature, such as Abdul et al., (2017), who produced GO-wood biochar nanocomposites at three temperatures (300 °C, 500 °C and 700 °C). They observed similar functional groups to those in our samples, mostly C- and O- containing groups. They also found that GO biochars had higher content, compared to the untreated ones. Moreover, they found that at 500 °C the percentage of C = O bonds increased by 27%-30%, but at 700 °C it decreased by 29%-31%, compared to the samples produced at 300 °C. Furthermore, Tang et al., (2015) produced GO coated wheat straw biochars at 600 °C and observed a significant increase on the oxygen based functional groups of the engineered biochar. Finally, Ghaffar and Younis (2014) used peanut shell biochars treated with GO at 300 °C and 500 °C. They observed a slightly different behavior, where GO treatment improved C-based functional groups, but higher temperature decreased them. Though, both GO treatment and pyrolysis temperature provided biochars that were significantly more effective adsorptive behavior for the removal of organics from water, compared to the untreated ones.

**Fig. 6:**
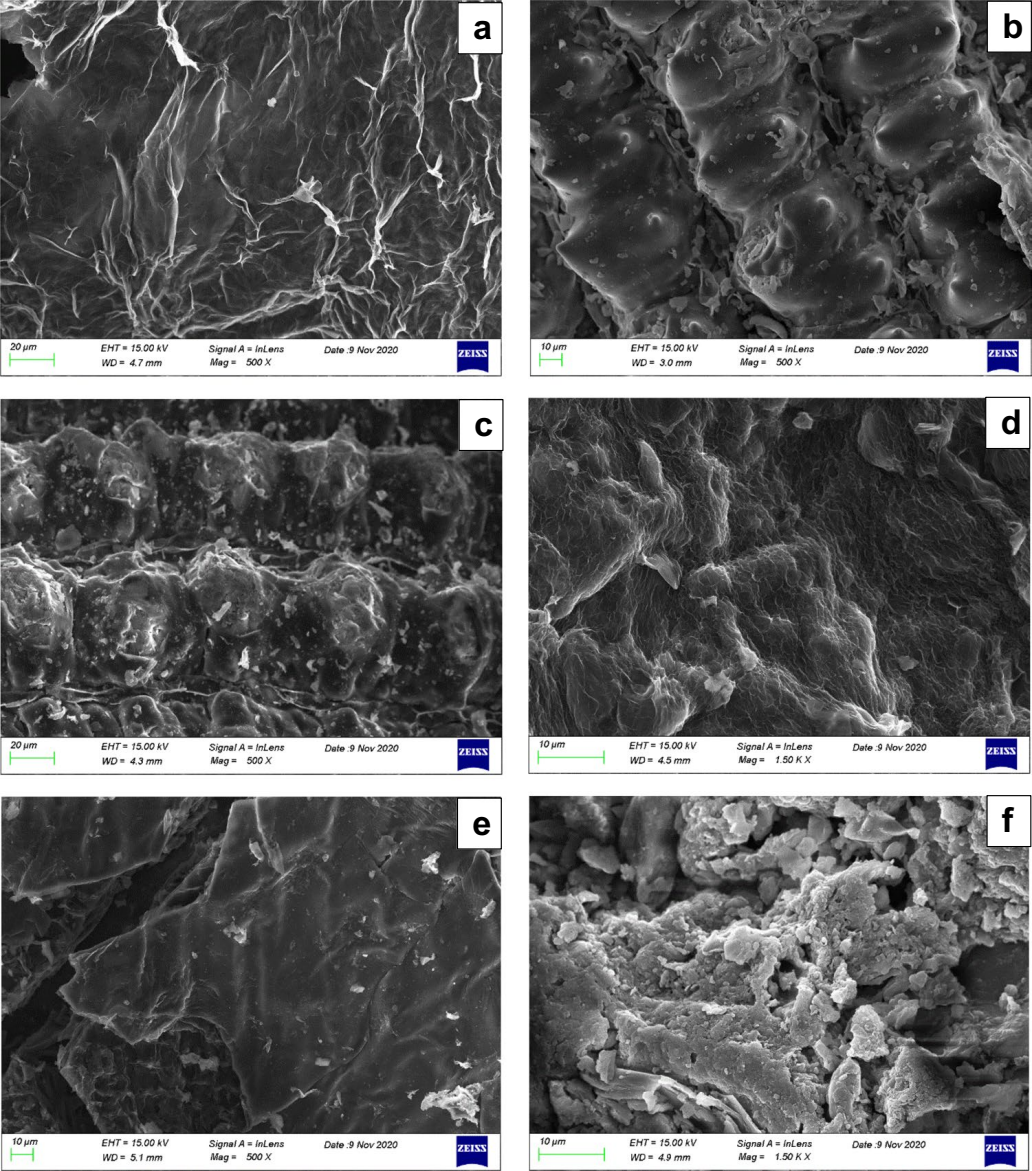

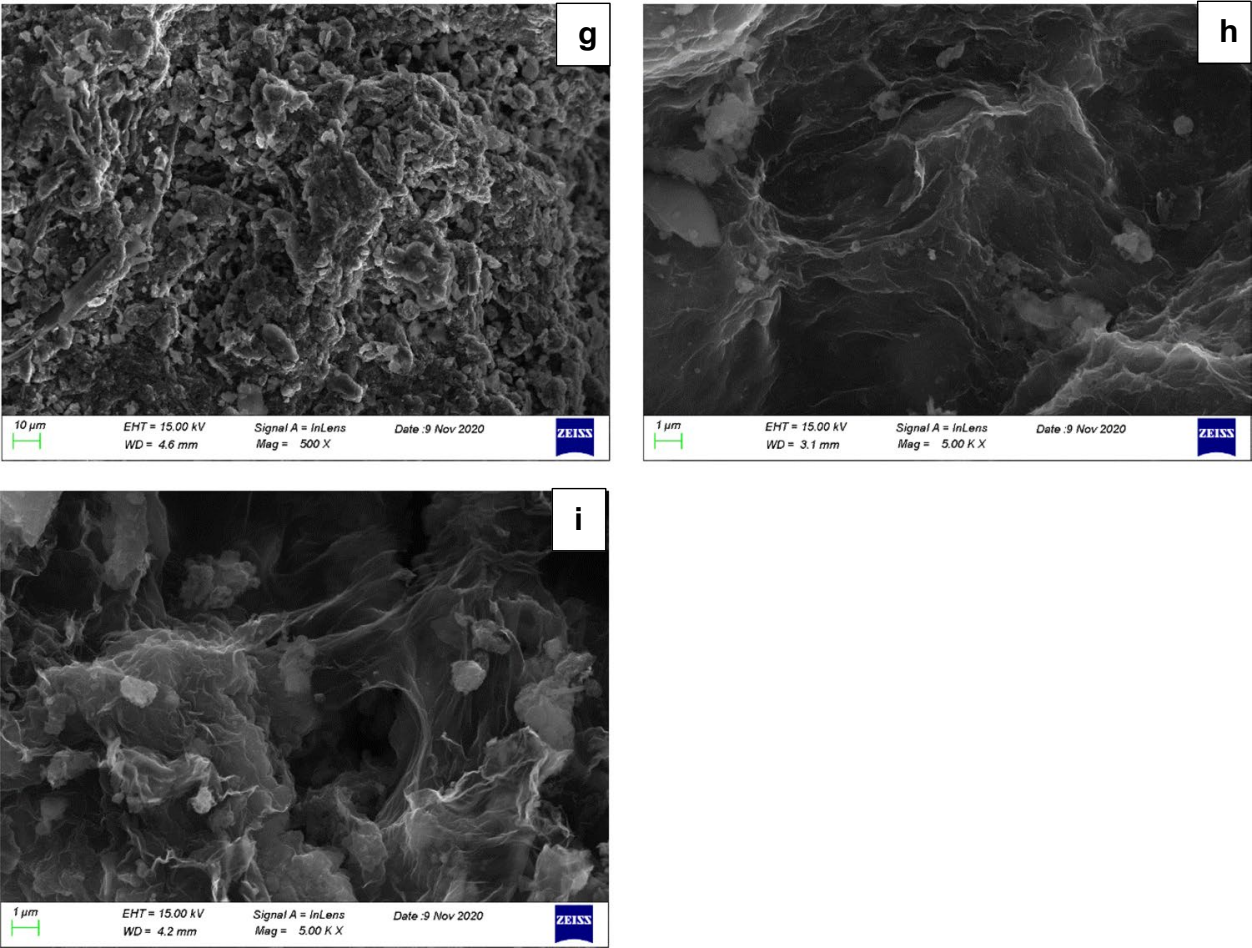
SEM analysis: **a**: GO, **b**: RH_400, **c**: RH600, **d**: RH_GO1_400, **e**: RH_GO1_600, **f**: SS_400, **g**: SS600, **h**: SS_GO1_400, **i**: SS_GO1_600

SEM images of the investigated samples are depicted in Fig. 6. Results showed that higher temperature (6b, 6f) led to more carbonized biochar, presenting a rougher and more porous surface (6c, 6 g), which agrees to the results obtained from the physicochemical characterization of the samples. The main goal of this analysis though was to investigate the success of GO implementation to the biomass and consequently to the produced functionalized biochars. Functionalization was clearly a success, which can be witnessed in Figs. 6d, e, h and i, where the net-like structure of GO (6a) got implemented on the pores of biochar surface, providing it with additional surface functional groups, as witnessed in the XPS analysis of the samples.

### Organic micro-pollutant adsorption experiments

**Fig. 7:**
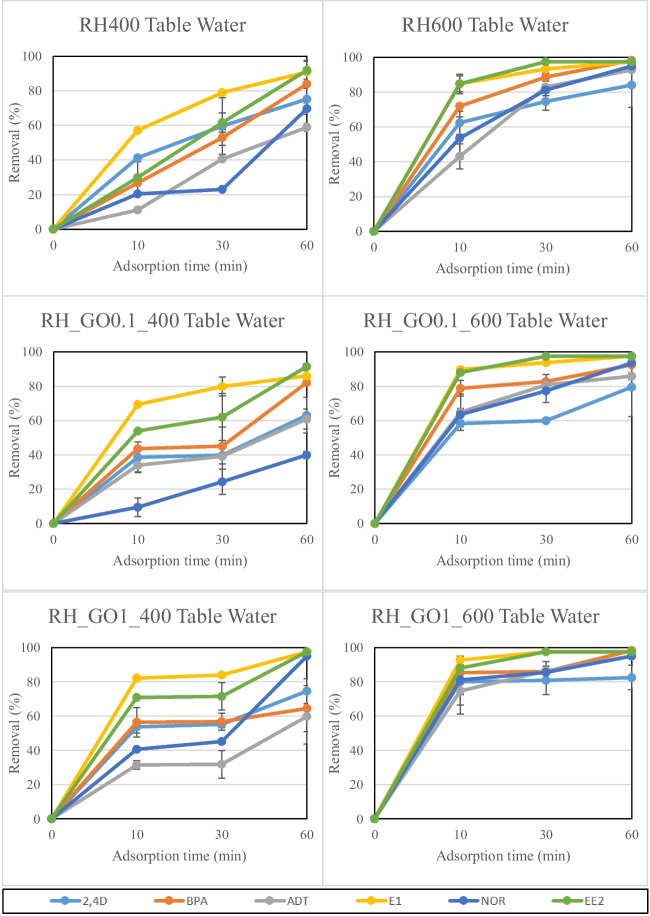
Organic micro-pollutant adsorption on RH biochars in table water. Error bars depict standard deviation of duplicate repetition

Figure 7 depicts the results concerning the adsorption of six organic micro-pollutants on conventional and GO-engineered RH biochars, in table water. Organic micro-pollutant removal was in the range of 58.9%-92.0% for RH400, 84.1%-98.3% for RH600, 39.9%-91.5% for RH_GO0.1_400, 79.5%-97.5% for RH_GO0.1_600, 59.8%-97.5% for RH_GO1_400 and 82.5%-98.3% for RH_GO1_600, in 60 min of adsorption time. These results indicate that biochars produced at 600 °C were more efficient concerning the adsorption of the six organic micro-pollutants by achieving higher removal rates (79.5%-98.3%), compared to biochars produced at 400 °C (39.9%-97.5%). This was expected and has been witnessed by several researchers before (Regkouzas & Diamadopoulos 2019; Tang et al. 2015), since higher pyrolysis temperature results into more stable, porous and organized C-structures, making the final product a better adsorbent. Moreover, GO functionalization provided better results in terms of adsorption time. Specifically, GO-functionalized biochars produced at 600 °C achieved > 70% pollutant removal by the first 30 min, while sample RH_GO1_600, which was the most effective of the biochars tested, achieved approximately > 74.7% pollutant removal by the first 10 min of adsorption time.

**Fig. 8:**
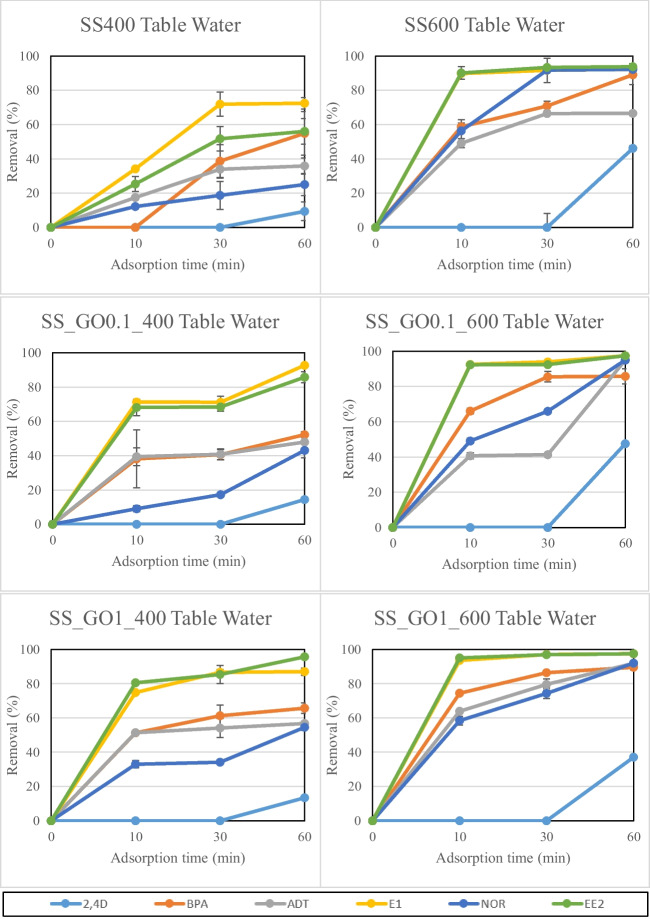
Organic micro-pollutant adsorption on SS biochars in table water. Error bars depict standard deviation of duplicate repetition

Figure 8 depicts the results concerning the adsorption of the investigated organic micro-pollutants on conventional and GO-engineered SS biochars, in table water. Removal was in the range of 9.4%-72.4% for SS400, 46.2%-93.8% for SS600, 14.4%-92.6% for SS_GO0.1_400, 47.5%-97.5% for SS_GO0.1_600, 13.4%-95.7% for SS_GO1_400 and 37.1%-97.5% for SS_GO1_600, in 60 min of adsorption time. The role of higher pyrolysis temperature for biochar production was even more highlighted by the SS biochar adsorption results, where samples produced at 600℃ showed higher adsorption rates (14.4%-97.5%) compared to those produced at 400℃ (9.4%-95.7%). Furthermore, GO functionalization was proved to be important for the SS samples too, where functionalized biochars could remove micro-pollutants faster and at higher rates compared to untreated biochars. Specifically, GO functionalized biochars at 600℃ achieved > 40% pollutant removal by the first 10 min of adsorption time for the majority of the investigated compounds. This was attributed to the additional and important surface C-based functional groups that GO provided to the integrated biochar samples, combined with its large surface area and porosity (Plaza et al. 2019). An important observation concerning the SS biochar adsorption results in table water was the significant difficulty concerning the removal of 2.4D. Removal rates for this compound were significantly low (9.4%-46.2%) and achieved only after 60 min of adsorption time. These results were attributed to the higher pH_PZC_ values of SS biochars (6.9–7.7) compared to RH biochars (4.4–7.2), which made it more difficult for 2.4D to get adsorbed. 2.4D has a pk_a_ value of 7.89, which is close to the pH_PZC_ median values of SS biochars. In our previous work, we observed similar trends and concluded that adsorption of 2.4D is less efficient when solution pH values and biochar pH_PZC_ values are equal or higher than the pk_a_ value of the pollutant (Regkouzas & Diamadopoulos 2019), leading to reduced adsorption efficiency and potential pore blockage (Xiong et al. 2021).

**Fig. 9:**
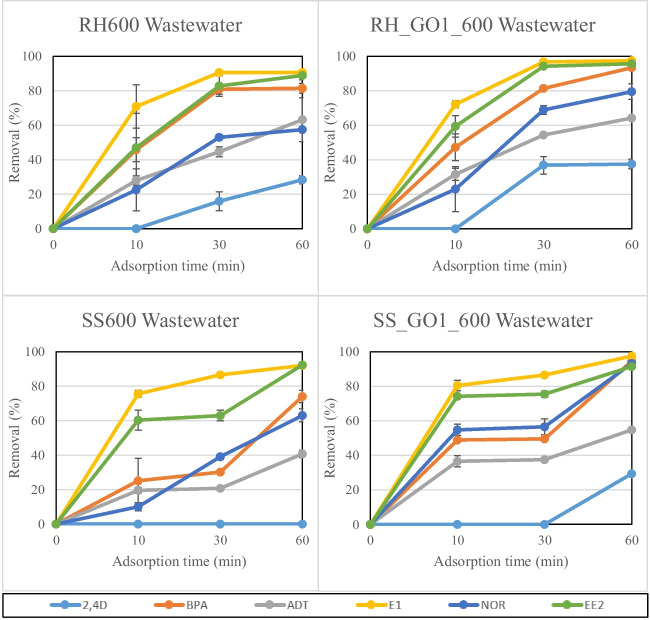
Organic micro-pollutant adsorption on RH and SS biochars in wastewater. Error bars depict standard deviation of duplicate repetition.

Figure 9 contains the results regarding the micro-pollutant adsorption on selected RH and SS biochars in wastewater. Experiments were performed on untreated and 1% GO-functionalized biochars at 600℃. Pollutant removal was in the range of 28.3%-97.5% for RH biochars and 0%-97.5% for SS biochars. Adsorption efficiency was higher for RH biochars, which is consistent with the table water adsorption results. Moreover, GO-functionalized biochars were also more effective compared to the untreated ones, leading to slightly higher pollutant removal rates. The two estrogens, E1 and EE2 were removed at the highest rates (88.9%-97.5%), followed by NOR, BPA and ADT, while 2.4D was the hardest compound to adsorb in this case too. 2.4D removal rates ranged between 0% and 37.6% in 60 min of adsorption time, where SS600 completely failed to adsorb the compound, even after 60 min of contact time. Apart from the parameters discussed in the SS biochar table water results, antagonistic interactions between the organic or inorganic compounds existing in the secondary effluent along with the investigated pollutants were probably another significant reason for the reduced adsorption rates of 2.4D (Kalderis et al. 2017). This phenomenon leads to biochar pore blockage and as a result, lower adsorption efficiency (Chen et al. 2021). For the same reasons, micro-pollutant removal from treated municipal wastewater was lower compared to the table water adsorption results, which was also witnessed in our previous work (Regkouzas & Diamadopoulos 2019).

The most likely involved mechanisms in the case of this study were pore-filling, which is strongly connected to biochar micro- and meso- porous nature formed in temperatures above 400℃ and is described by high and fast pollutant removal rates (Regkouzas & Diamadopoulos 2019), electrostatic interactions with ionic compounds or chemisorption, which created strong electrostatic interaction between biochar surface and the polar nature of the investigated pollutants (Abbas et al. 2018), along with hydrophobic reactions, which combine partitioning and hydrophobic attraction attributed to the non-carbonized or partially carbonized fractions on biochar surface and playing a significant role concerning hydrophobic compound adsorption (Ambaye et al. 2021) .

A significant matter concerning the biochar research community is exhausted biochar reuse. Biochars that have been previously used for adsorption purposes usually present lower adsorption efficiency when used in consecutive adsorption experiments concerning either organic or inorganic pollutants, which creates the need for proper reuse strategies of the material (Gao et al. 2022). Agronomic application of exhausted biochar as soil amendment seems to be the most sustainable solution, as it can offer beneficial properties to the amending soil and cultivation, such as nutrient supplementation, carbon mitigation, increase of the water holding capacity, enhancement of soil microbial activity and several other properties (Mosa et al. 2018, 2020). In order to assess EC loaded exhausted biochar, further experimental work is needed, but based on previous works, the majority of the adsorbed ECs would probably remain in non-leachable form in biochar structure without posing a threat to the applied soil (Mer et al. 2021). Other works have shown that exhausted biochar could also be reused in the building industry as cement additive, leading to a significant carbon footprint decrease of these materials (Legan et al. 2022; Danish et al. 2021).

## Conclusion

The results of this work showed that the main parameter that affected biochar physicochemical structure and composition was biomass type, followed by pyrolysis temperature and GO functionalization. RH is an agronomic lignocellulotic biomass and biochars produced from it showed significantly more favorable properties than SS, which is an anthropogenic waste, containing various burdening compounds in its structure, such as high inorganic content, which works negatively for many of biochar properties. Moreover, higher pyrolysis temperature also improved biochar, by increasing the carbon content, S_BET_ and surface functional groups, thus presenting a more aromatic structure. The metal content of biochars was within the permissible limits set by the European Union and in combination with our previous work, it could be suggested that RH and SS biochar could be safely used for environmental applications. FTIR and XPS analyses showed an abundance and variety of elements and functional groups on biochar surface, where SS biochar showed greater elemental variety, but RH biochars showed better distributed C-based functional groups, which is very important for their use as adsorptive materials. This was confirmed by the organic micro-pollutant adsorption results, where RH biochars were better than SS biochars as adsorbents, achieving higher pollutant removal rates in less contact time. Based on the results of this work, we conclude that the best biochar produced was RH_GO1_600, where the abundance and variety of its C-based functional groups made it a successful adsorbent for organic micro-pollutants from water and treated municipal wastewater.

## Supplementary Information

Below is the link to the electronic supplementary material.

Supplementary file1 (DOCX 562 KB)

## Data Availability

On inquiry, the data presented in this study is available from the authors.

## Abbreviations

ADT: Androsterone
GO: Graphene Oxide
BPA: Bisphenol A
E1: Estrone
EE2: 17α-Ethinylestradiol
NOR: 19-Norethindrone
RH: Rice Husks
RH400: Biochar from Rice Husks, produced at 400 °C
RH600: Biochar from Rice Husks, produced at 600 °C
RH_GO0.1_400: Biochar from Rice Husks, with 0.1% GO suspension, produced at 400 °C
RH_GO0.1_600: Biochar from Rice Husks, with 0.1% GO suspension, produced at 600 °C
RH_GO1_400: Biochar from Rice Husks, with 1% GO suspension, produced at 400 °C
RH_GO1_600: Biochar from Rice Husks, with 1% GO suspension, produced at 600 °C
SEM: Scanning Electron Microscope
SPME: Solid Phase Micro-Extraction
SS: Sewage Sludge
SS400: Biochar from Sewage Sludge, produced at 400 °C
SS 600: Biochar from Sewage Sludge, produced at 600 °C
SS _GO0.1_400: Biochar from Sewage Sludge, with 0.1% GO suspension, produced at 400 °C
SS _GO0.1_600: Biochar from Sewage Sludge, with 0.1% GO suspension, produced at 600 °C
SS_GO1_400: Biochar from Sewage Sludge, with 1% GO suspension, produced at 400 °C
SS_GO1_600: Biochar from Sewage Sludge, with 1% GO suspension, produced at 600 °C
WWTP: Wastewater Treatment Plant
2-4D: 2, 4-Dichlorophenol
