# Supplementary material for: Production and characterization of graphene oxide-engineered biochars and application for organic micro-pollutant adsorption from aqueous solutions
Source: Environ Sci Pollut Res Int. 2023 Jul 11;30(37):87810–29. doi: 10.1007/s11356-023-28549-y (PMC10406730; doi:10.1007/s11356-023-28549-y)
Supplement: Supplementary file 1 — Supplementary file1 (DOCX 562 KB) [file 11356_2023_28549_MOESM1_ESM.docx]

**Table S1:** Table water and wastewater sample characterization

| **Table water** | | **Wastewater** | |
| --- | --- | --- | --- |
| **pH** | 7.9 | **pH** | 7,8 |
| **EC** | 251μS cm^-1^ | **BOD** | 6mg L^-1^ |
| **Dry Residue** | 162mg L^-1^ | **COD** | 13-15mg L^-1^ |
| **Total Hardness** | 128 mg L^-1^ CaCO_3_ | **TOC** | 5,3ppm |
|  |  | **TSS** | 7mg L^-1^ |

**Figure S1:** Deconvoluted N1s peaks of S1a: SS600, S1b: SS_GO1_600, S1c: RH600, S1d: RH_GO1_600.

**Table S1:** % component concentrations of N1s peaks.

| SS600 | | | |
| --- | --- | --- | --- |
| Peak | **Eb (eV)** | **% Component C** | **Assignement** |
| N1s | 398.74 | 38.72 ± 1.95 | Pyrrole (N-C) |
| N1s | 400.74 | 61.28 ± 1.95 | Amide (N-C=O) |
| N1s | 405.61 | 0.00 ± 0.00 | NO2 (C-NO2) |
| SS_GO1_600 | | | |
| Peak | **Eb (eV)** | **% Component C** | **Assignement** |
| N1s | 398.68 | 45.54 ± 2.51 | Pyrrole (N-C) |
| N1s | 400.65 | 46.53 ± 2.20 | Amide (N-C=O) |
| N1s | 405.48 | 7.93 ± 0.80 | NO2 (C-NO2) |
| RH600 | | | |
| Peak | **Eb (eV)** | **% Component C** | **Assignement** |
| N1s | 398.80 | 32.23 ± 5.13 | Pyrrole (N-C) |
| N1s | 400.69 | 67.77 ± 5.13 | Amide (N-C=O) |
| RH_GO1_600 | | | |
| Peak | **Eb (eV)** | **% Component C** | **Assignement** |
| N1s | 398.78 | 31.33 ± 3.70 | Pyrrole (N-C) |
| N1s | 400.66 | 59.10 ± 4.51 | Amide (N-C=O) |
| N1s | 405.59 | 9.57 ± 2.73 | NO2 (C-NO2) |
